# The association between human papillomavirus 16 and esophageal cancer in Chinese population: a meta-analysis

**DOI:** 10.1186/s12885-015-1096-1

**Published:** 2015-03-05

**Authors:** Shao-Kai Zhang, Lan-Wei Guo, Qiong Chen, Meng Zhang, Shu-Zheng Liu, Pei-Liang Quan, Jian-Bang Lu, Xi-Bin Sun

**Affiliations:** Department of Cancer Epidemiology, Henan Cancer Hospital, Henan Office for Cancer Control and Research, Affiliated Cancer Hospital of Zhengzhou University, Zhengzhou, China

**Keywords:** Esophageal cancer, Genotype, Human papillomavirus, Meta-analysis, China

## Abstract

**Background:**

The role of human papillomavirus (HPV) in the development of esophageal cancer remains controversial. Our study aims to test the association between HPV 16 infection and esophageal cancer in China, providing useful information on this unclear association in Chinese population.

**Methods:**

Studies on HPV infection and esophageal cancer were identified. A random-effects model was used to calculate the odds ratios (ORs) and corresponding 95% confidence intervals (CIs) comparing cases with controls.

**Results:**

A total of 1442 esophageal cancer cases and 1602 controls from 10 included studies were evaluated to estimate the association between HPV 16 infection and esophageal cancer risk. The ORs for each case–control studies ranged from 3.65 (95% *CI*: 2.17, 6.13) to 15.44 (95% *CI*: 3.42, 69.70). The pooled estimates for OR was 6.36 (95% *CI*: 4.46, 9.07). In sensitivity analysis, the estimates for OR ranged from 5.92 (95% *CI*: 4.08, 8.60) to 6.97 (95% *CI*: 4.89, 9.93).

**Conclusions:**

This study indicates that HPV-16 infection may be a risk factor for esophageal cancer among Chinese population, supporting an etiological role of HPV16 in this malignancy. Results in this study may have important implications for esophageal cancer prevention and treatment in China.

## Background

Esophageal cancer is the eighth most common malignancy worldwide and a majority of cases show poor prognosis in clinical practice [1]. Based on IARC statistics, there are 456,000 new cases (3.2% of the total) and 400,000 deaths (4.9% of the total) in 2012 [1]. Globally, around 80% of the cases worldwide occur in less developed regions. China has a high burden of esophageal cancer. The incidence and mortality of esophageal cancer in China were 22.4/100000 and 16.77/100000 [2], respectively. It is becoming a substantial medical and public health challenge in China.

In the past few decades, many risk factors for esophageal cancer have been investigated, including tobacco smoking, alcohol drinking, dietary and micronutrient deficiency, high temperature of beverage and food consumption, poverty and history of head and neck cancer [3]. However, the etiology of esophageal cancer is still unclear. In China, infectious agents contributed more than one quarter of the overall cancer cases [4]. The role of infectious agents in esophageal carcinogenesis has also been suggested as either direct carcinogens or promoters.

Human papillomavirus (HPV) has been suggested as a distinct possible cause of esophageal cancer. To date, more than 140 HPV genotypes have been recognized and subdivided into cutaneous and mucosal HPV types. Based on the oncogenicity, HPV are classified into high-risk and low-risk types. High-risk HPV 16 infection is more prevalent than any other high-risk HPV type in most regions of the world [5]. As the upper gastrointestinal tract might be exposed to HPV through oral transmission, infection with oncogenic HPV as a contributor to esophageal cancer was hypothesised over three decades ago [6].

The role of HPV in the development of esophageal cancer remains controversial. The prevalence of HPV infection in esophageal lesion or carcinomas varied largely in different studies [7]. Syrjanen et al. summarized the HPV prevalence of any type in esophageal cancer and reported that the mean prevalence of HPV was 29.0%, ranging from 0% to 78% [8]. Some studies reported that the prevalence of HPV were relatively high in high risk areas of esophageal cancer in the world [9]. Studies also showed a positive correlation between HPV 16 and esophageal cancer [10-12]. However, the association between HPV 16 infection and esophageal cancer in Chinese population has not yet been assessed clearly. Establishing the relationship between HPV 16 infection and esophageal cancer may improve our understanding of its role and give some clues of immune efficacy of HPV vaccine for esophageal cancer, which have been successfully applied on cervical cancer. Therefore, we conducted this study to assess the association between HPV 16 infection and risk of esophageal cancer in China, providing useful information on this unclear association in Chinese population.

## Methods

### Literature search

A systematic search was conducted to identify relevant articles, using MEDLINE (via PubMed), Excerpta Medica database (EMBASE) for English language, and using Chinese National Knowledge Infrastructure and Wanfang Data Knowledge Service Platform for Chinese language. Date of the literature was specified between 1 Jan 2005 and 1 July 2014. The search strategy was verified by a medical reference librarian and research articles were selected using the following keywords: human papillomavirus, papillomavirus infections, (o) esophageal neoplasms, (o) esophageal cancer, and (o) esophageal carcinoma. The search strategy was adapted for each database in order to maximize the ability to identify eligible studies. Reference lists of the included studies and published meta-analyses on related topics were also screened for additional studies.

### Eligible criteria

Two authors independently reviewed the identified relevant articles and judged whether they met the inclusion criteria for meta-analysis. Uncertainties and discrepancies were resolved by consensus after discussion with a senior author. The meta-analysis included studies in adults meeting the following criteria: (1) case–control studies or cohort studies; (2) studies detected HPV DNA in the tissues of subjects; (3) samples of control group must be taken from normal population without esophageal cancer; (4) studies explicitly provided the information on HPV DNA detection method. HPV DNA must be tested either by polymerase chain reaction (PCR)-based methods, including broad-spectrum PCR primers, type-specific PCR primers, or a combination of both kinds of primers; (5) necessary data could be directly extracted or calculated from the original article; (6) studies were peer-reviewed publications with HPV prevalence data from a minimum of 30 cases of esophageal cancer; and (7) studies conducted in the Chinese population. If the study was reported in duplication, the one published earlier or provided more detailed information was included. Review articles and editorials were included if they contained original data. Abstracts were excluded.

### Data extraction

Two authors performed the data extraction from each article and discrepancies were resolved by consensus. For studies meeting the inclusion criteria, a standard data extraction form was used to extract the following data: general information, including name of first author, year of publication, geographical areas of the study origin; numbers of cases/controls and HPV positive cases/controls; HPV detection method; and types of specimen (paraffin-embedded fixed biopsies (PE), fresh or frozen biopsies (FF)).

### Statistical analyses

In this meta-analysis, the association between HPV infection and cancer risk was estimated by means of odds ratios (ORs) and corresponding 95% confidence intervals (CIs) comparing cases with controls through the method of DerSimonian and Laird using the assumptions of a random-effects model [13]. For subjects with multiple HPV types infection (including HPV 16), the multiple HPV types were separated into different types and the HPV 16 type-specific prevalence represents types for subjects with either single HPV 16 infection or multiple HPV 16 infection. When multiple control groups were studied, we selected the group of subjects providing normal mucosal biopsies.

Heterogeneity between eligible studies were assess by *I*^*2*^ (values of 25%, 50% and 75% corresponding to low, moderate and high degrees of heterogeneity, respectively) and Cochrane *Q* test (*P* < 0.10 indicated a high level of statistical heterogeneity) [14]. Stratified pooled analyses were subsequently carried out according to the geographical areas of the study origin, publication years, HPV detection method and types of specimen. Sensitivity analysis was conducted to assess the influence of each individual study on the strength and stability of the meta-analytic results. Each time, one study in the meta-analysis was excluded to see its impact on the combined effect size. Publication bias was assessed with Begg’s and Egger’s tests and also by examining for irregularities in funnel plots demonstrating the relationship between the individual log ORs and their standard errors [15,16]. In addition, a cumulative meta-analysis was conducted to investigate the cumulative evidence at the time that each study was published to show the trend of results over time.

In this study, meta-analyses were performed using STATA version 12 for Windows (StataCorp LP, College Station, TX, USA). A two-tailed P < 0.05 was considered statistically significant.

## Results

**Figure 1:**
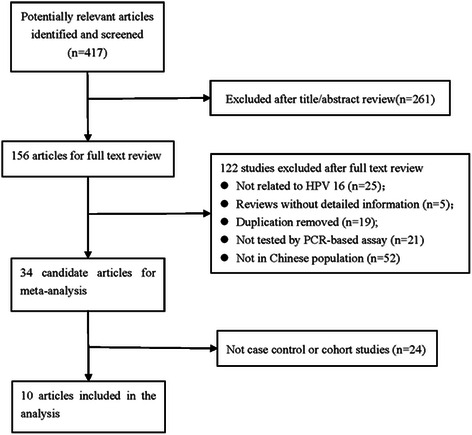
**Flow diagram for the selection of included studies.**

Figure 1 shows the flow diagram for the selection of included studies. The systematic literatures search yielded 417 articles relevant to the topic using different combination of key words, of which 156 were considered as having potential value and the full texts were retrieved for detailed evaluation. One hundred and twenty-two of the 156 articles were subsequently excluded from the meta-analysis. The majority of the reasons for exclusion were: studies not conducted in Chinese population, studies not related to HPV 16 or studies not tested by PCR-based assay. Duplicated studies and reviews without detailed information were also excluded. Furthermore, twenty-four studies were also excluded as they were not case control or cohort studies. At last, we included 10 eligible studies in the meta-analysis [17-26].

Individual characteristics of the included 10 studies were summarized in Table 1. The included studies were conducted during 2007–2014. In total, 1442 esophageal cancer cases and 1602 controls were evaluated to estimate the association between HPV 16 infection and esophageal cancer risk in the 10 included studies. Of these studies, 3 were conducted in Henan province, and other studies were conducted in provinces of Xinjiang (3), Shandong (1), Shaanxi (1), Chongqing (1) and Guangdong (1). The most used types of esophageal specimens to test HPV DNA status were PE, which accounted for 70% of the included studies, and the other 30% specimens were FF. The HPV detection region of these studies were L1 (50%) or HPV E6 (50%). In the eligible studies, the HPV 16 prevalence ranged from 0.23 to 0.69 in cases. With respect to controls, the HPV 16 prevalence was mainly from 0.02 to 0.22, except one study in which the prevalence was 0.37, much higher than other studies.

**Table 1:** **Characteristics of 10 studies included in the meta-analysis**

Reference	Region	Number of HPV 16 positive in cases	HPV16 prevalence in cases	Number of HPV 16 positive in controls	HPV 16 prevalence in controls	Types of specimen^1^	HPV detection method
Liu et al. [17]	Chongqing	43/112	0.38	4/74	0.05	FF	L1
He et al. [18]	Henan	56/110	0.51	7/45	0.16	FE	E6
Chen et al. [19]	Xinjiang	34/80	0.43	11/80	0.14	FE	E6
Liu et al. [20]	Shaanxi	35/69	0.51	2/32	0.06	FE	E6
Han and Chen [21]	Shandong	121/204	0.59	12/102	0.12	FE	L1
Guo et al. [22]	Henan	70/300	0.23	21/900	0.02	FF	L1
Hu et al. [23]	Xinjiang	82/200	0.41	24/150	0.16	FE	E6
Liu et al. [24]	Henan	54/78	0.69	11/30	0.37	FF	L1
Zhang et al. [38]	Guangdong	62/106	0.58	22/100	0.22	FE	E6
Cui et al. [26]	Xinjiang	53/183	0.29	8/89	0.09	FE	L1

^1^FF: Fresh-Frozen; PE: Paraffin-Embedded.

**Figure 2:**
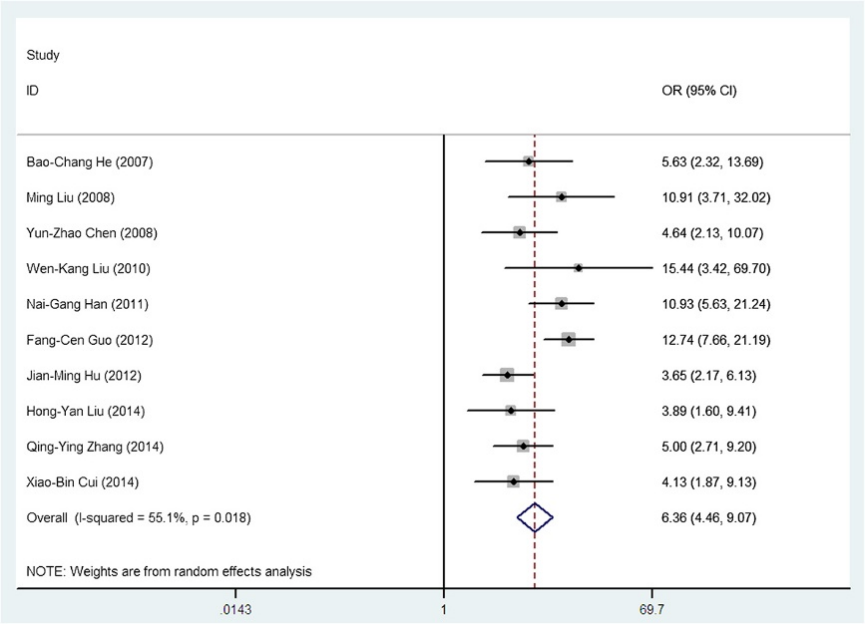
**Forest plot for meta-analysis of the association of HPV with esophageal cancer in 10 case–control studies.**

Based on the heterogeneity test, there was a moderate heterogeneity between included studies (*Q* test *P*_heterogeneity_ < 0.001, *I*^*2*^ = 55.1%). Therefore, the random-effects model was chosen to evaluate the pooled ORs. Individual and pooled OR estimates derived from a random effect model analysis were illustrated in the Forest plot. As shown in Figure 2, the ORs for each case–control studies ranged from 3.65 (95% CI: 2.17, 6.13) to 15.44 (95% *CI*: 3.42, 69.70). The pooled estimates for OR was 6.36 (95% *CI*: 4.46, 9.07), indicating a significant association between HPV 16 infection and esophageal cancer.

Results stratified by geographical areas of the study origin, publication years, types of specimen and HPV detection method were presented in Table 2. Based on these information, we can observed that the point estimate for pooled OR was higher for studies conducted in Northern China. Similarly, studies which HPV DNA extracted from FF tissues, or which detected gene from L1 region of HPV were generally revealed to be higher point estimate.

**Table 2:** **Meta-analysis of the association between HPV 16 and esophageal cancer by different variables**

Variable	Number of studies	OR (95% CI)	Heterogeneity	
			*P*for Q test	*I*^*2*^(%)
**Region**				
North	4	8.15 (4.79, 13.86)	0.084	54.9
Northwest	4	4.36 (2.95, 6.44)	0.357	7.2
South	2	6.50 (3.12, 13.55)	0.209	36.5
**Year**				
2005-2009	4	6.65 (4.07, 10.86)	0.384	1.60
2010-2014	6	6.03 (3.72, 9.77)	0.004	70.7
**Specimen** ^**(1)**^				
PE	7	5.48 (3.88, 7.52)	0.169	34.0
FF	3	8.48 (3.99, 9.41)	0.072	61.9
**HPV detection method**				
L1	5	7.87 (4.71, 13.16)	0.052	57.3
E6	5	4.68 (3.40, 6.44 )	0.473	0.00

^(1)^FF: Fresh-Frozen; PE: Paraffin-Embedded.

**Figure 3:**
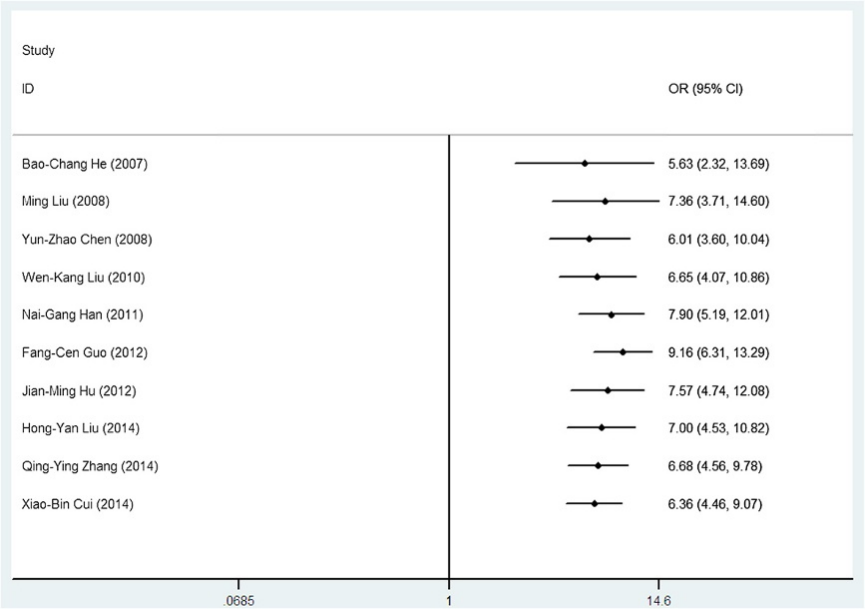
**Cumulative meta-analysis of case control studies for the evidence of association between HPV and esophageal cancer.**

Figure 3 showed the results of cumulative random-effects meta-analysis of the 10 studies. All studies revealed a positive association between HPV 16 and esophageal cancer. There was a weaker association in the earliest study conducted in 2007 (*OR* = 5.63, 95% *CI*: 2.32, 13.69), compared to the latest study in 2014 with a cumulative estimate of 6.36 (95% *CI*: 4.46, 9.07). The confidence interval for the summary estimate of the cumulative studies decreased with time of studies (Figure 3).

**Figure 4:**
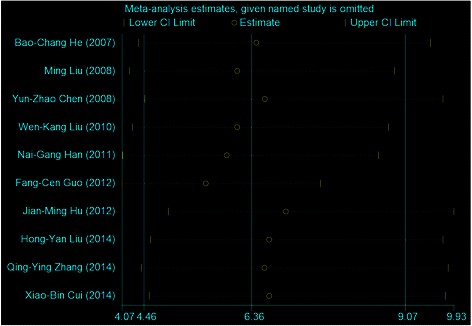
**Sensitivity analysis for individual studies on the summary effect.**

To address the potential bias due to the quality of the included studies, we performed the sensitivity analysis by calculating pooled OR again when omitting one study each time. Figure 4 showed the results of sensitivity analysis. The estimates for OR ranged from 5.92 (95% *CI*: 4.08, 8.60) to 6.97 (95% CI: 4.89, 9.93). Results did not show significant differences when any study was omitted, which indicated that each single study did not influence the stability of the association between HPV 16 and esophageal cancer.

The funnel plot did not show evidence of asymmetry (Not shown). According to Begg’s and Egger’s test, there was no evidence of publication bias (Begg *P* = 0.721, Egger *P* = 0.878).

## Discussion

To our knowledge, this is the first meta-analysis that aimed to explore the association between HPV 16 and esophageal cancer in Chinese population, including 1442 esophageal cancer cases and 1602 controls. This meta-analysis highlights an over sixfold increased risk of esophageal cancer in the presence of HPV 16 infection, providing a strong evidence to date of a potential role for HPV 16 in the etiology of esophageal cancer in Chinese population.

Results in our meta-analysis are consistent with other studies. Yong et al. reported an OR of 3.55 (95% CI: 2.05, 6.14) between HPV 16 infection and esophageal cancer. Li et al. reported a similar OR of 3.52 (95% CI, 2.04–6.07) [11]. Hardefeldt et al. also investigated this issue and acquired an OR of 2 · 35 (95% CI: 1.73, 3.19) [12]. Although all these studies showed an increased risk associated with HPV 16 infection, the pooled OR (6.36, 95% *CI*: 4.46, 9.07) in our meta-analysis is relatively higher than other studies. This is not surprising if we consider the inclusion criteria of this meta-analysis. We only included studies provided the information of HPV DNA tested by PCR-based assay which is more sensitive than other methods, such as in situ hybridization histochemistry. Even more importantly, samples of control group were taken from normal population without esophageal cancer. Studies using other normal participants had a higher risk than studies with adjacent normal mucosa as the normal control [27]. This was possibly due to paraneoplastic impairment of the local oesophageal mucosal immunity and using adjacent normal mucosa as controls could not exclude this effect. Therefore, independent normal controls should yield a more accurate estimate of the risk.

Some factors might contribute to the variability of results on evaluating the association of HPV 16 infection and esophageal cancer. In our study, stratified analyses were performed according to geographical areas of the study origin, publication years, HPV detection method and types of specimen. Based on the results, we found that samples from FF tissues had a higher point estimate of OR than samples from PE tissues. This is mainly due to the DNA degradation in PE tissue [28]. We also found that the DNA source of HPV can also affect the estimate of risk. In the future study investigating the relationship between type specific HPV and esophageal cancer, these factors should be considered to acquire a more realistic result.

Our results may have important implications for esophageal cancer prevention and treatment. To date, two vaccines have been developed and approved for use against HPV. Gardasil is a quadrivalent vaccine that targets HPV-6, −11, −16, and −18. The bivalent vaccine Cervarix targets HPV-16 and −18. Recently, the success of the prophylactic immunization campaign for cervical cancer has attracted a lot of interest in preventable HPV related cancers, including esophageal cancer. However, the association between HPV and esophageal cancer is controversial since it was first reported by Syrjänen [29]. Therefore, an established association between type-specific HPV infection and esophageal cancer is essential for HPV screening and vaccination policies. Results in our study support that HPV vaccine could benefit populations at high risk of esophageal cancer in China, which is a good sign for alleviating the esophageal cancer burden in China. Although we expect for improved therapeutic modalities for esophageal cancer, perhaps the greatest potential lies in the ability to prevent the development of esophageal cancer, including the widespread HPV vaccination. Despite esophageal cancer could be caused by different factors, we can totally prevent HPV-related esophageal cancer through vaccine. In addition, some studies have suggested that patients with HPV-positive cancers had a better prognosis than patients with HPV-negative cancers, such as head and neck cancer [30-33]. Esophagus can be infected with HPV in the same way as head and neck cancer. It is thus considered that esophagus might have a similar association and clinical characteristics. As the first step, our results have demonstrated the strong association between HPV 16 and esophageal cancer. Another step is to investigate the prognostic value of the HPV 16 status in patients with esophageal cancer in Chinese population. Studies have reported that the patients with HPV-associated carcinoma have an improved prognosis than those with HPV-negative tumors, such as head and neck cancer [34,35]. Consistent with these results, Cao et al. have demonstrated that HPV-associated esophageal cancer treated with surgery achieves a superior outcome compared with HPV-negative esophageal cancer [36]. However, there were also studies which reported that the presence of HPV infection in esophageal cancer may be a factor indicating a relatively poor prognosis [37,38]. The relationship between the immune system, HPV status, and outcome of esophageal cancer is an interesting research area. Such research will have significant implication for esophageal cancer treatment.

This meta-analysis shows minimal heterogeneity or publication bias, and that no single study affects the summary effect significantly. The pooled result is further validated by the stratified analysis. All these strengthen the finding of the association between HPV 16 and esophageal cancer. However, there are still some limitations that should be addressed. First of all, due to lack of detailed information on confounders such as age, gender, smoking, assumption of alcohol, which were also risk factors of esophageal cancer, we could not adjust for these confounders which may affect the association between HPV 16 and esophageal cancer. Further studies should focus on this issue and provide more information on type-specific HPV risk on esophageal cancer. Secondly, we cannot exclude the effect of contamination of samples in this study which can directly affect the detection of HPV 16. Roden et al. reported that dehydrated HPV could maintain 100% infectivity for one day [39]. Ferenczy et al. and Strauss et al. found that HPV DNA existed on fomites and various medical surfaces [40,41], which should be responsible for sample contamination [39]. Despite our efforts to control the impact of contamination, this was difficult as many studies did not report on these issues. Future studies on the association between HPV and esophageal cancer should avoid contamination and record the quality control measures, which will help us to understand the role of HPV in esophageal cancer. Thirdly, we mainly focused on the association between HPV 16 and esophageal cancer in this study and did not investigate the overall HPV risk on esophageal cancer which is essential for esophageal cancer prevention in China. We will conduct further research to obtain more information.

## Conclusions

This study indicates that HPV-16 infection may be a risk factor for esophageal cancer among Chinese population, supporting an etiological role of HPV16 in this malignancy. We believe that this is a major step forward for this controversial issue and our results may have significant implications for esophageal cancer prevention in China. Further studies are needed to elucidate the role of HPV in esophagus carcinogenesis with careful consideration of study design and laboratory detection method, providing more accurate assessment of type specific HPV risk on esophageal cancer.
